# Developing Midlife Metacognitive Strategy Training (M-MCST) program: A feasibility study with middle-aged adults to evaluate the design, implementation, and impact of a novel program in fostering healthy cognitive aging

**DOI:** 10.1371/journal.pone.0358223

**Published:** 2026-09-25

**Authors:** Aysha Rooha, Sinchana Swamy, Nidhi Lalu Jacob, Gagan Bajaj, Chanchal Chaudhary, Vinitha Mary George, Jayashree S. Bhat

**Affiliations:** 1 Department of Audiology and Speech Language Pathology, Kasturba Medical College Mangalore, Manipal Academy of Higher Education, Manipal, India; 2 Department of Audiology and Speech Language Pathology, National Institute of Speech and Hearing, Trivandrum, India; 3 Department of Audiology and Speech Language Pathology, Nitte Institute of Speech and Hearing, Deralakatte, Mangalore, Karnataka, India; Ankara University Faculty of Medicine: Ankara Universitesi Tip Fakultesi, TÜRKIYE

## Abstract

**Background:**

Metacognitive skills mediate the role of cognitive reserves in aging and facilitate effective cognitive abilities. Metacognitive deficits may precede cognitive impairments, making it important to optimize metacognition as early as middle adulthood to promote cognitive communicative well-being. Thus, the present study aimed to develop and evaluate the feasibility of a novel metacognitive strategy training (MCST) program with neurotypical middle-aged adults (MAAs).

**Method:**

This study aligns with the initial stage of the National Institutes of Health (NIH) Stage Model for Behavioral Intervention Development. A modified nominal group technique was employed, involving a panel of five healthcare professionals to draft the strategy and execution-related aspects of the M-MCST. The feasibility of the developed protocol was then tested with twenty neurotypical middle-aged adults (MAAs) (Experimental group (EG)- 10; Control group (CG)- 10), on parameters such as recruitment, retention, adherence, changes in their cognitive performance, absolute self-estimation, and self-perception of performance using the Contextual Memory Test (CMT). Additionally, the EG participants provided reflections on the program's design, implementation, and impact to gauge participant acceptability of intervention.

**Results:**

The MCST comprises seven sessions incorporating the adapted ‘stop-think-plan-do-check’ metacognitive strategy, client-centered personalized goal selection, and a daily diary method for tracking progress. Feasibility testing revealed 100% recruitment, retention, and protocol adherence along with significant improvements in CMT performance, absolute self-estimation discrepancy, and self-perception of performance among trained MAAs. The participants found the design and implementation aspects of the training program to be promising.

**Conclusion:**

The MCST program guides healthcare professionals in the use of metacognitive strategy training with neurotypical adults, embedding it within everyday tasks. It incorporates mechanisms for monitoring strategy use, error awareness, and self-reflection through daily diaries. The findings demonstrate that the program is feasible and acceptable to MAAs and shows potential benefits for improving performance on the CMT and related metacognitive functioning. These findings support further evaluation of the program in large-scale efficacy trials and future investigations examining its potential contribution to broader constructs, such as cognitive reserve and healthy cognitive aging.

## Introduction

Within the realm of cognitive sciences, research has increasingly focused on enhancing cognitive reserves to counteract age-related cognitive disorders such as mild cognitive impairment and dementia. Cognitive reserve, defined as the brain’s ability to withstand neuropathological damage, is believed to be strengthened through experience-induced neuronal changes resulting from physically and mentally challenging activities [1]. Stern conceptualizes cognitive reserve as an active process in which the brain copes with damage by leveraging pre-existing cognitive strategies or recruiting compensatory neural networks [2]. Variables that influence cognitive reserves have been characterized as static and dynamic measures [3]. While occupational attainment and years of education tend to become static overtime, other factors such as general intellectual functions, literacy and engagement in cognitive activities serve as dynamic variables that continue to shape cognitive reserve across the lifespan [3]. Engagement across multiple life domains through these dynamic measures supports reserve-building as an ongoing adaptive process [2], with participation in cognitive tasks, an important example within leisure activities, contributing to both the current and retrospective aspects of cognitive reserve (2CR) [4]. The scaffolding theory of aging and cognition also supports this understanding that cognitive training enhances the development of compensatory recruitment of additional brain circuitry to shore up function, thereby conferring protection on cognitive function [5]. Consequently, numerous cognitive training programs targeting neurotypical adults in midlife and later adulthood have been developed and implemented, with demonstrated success in enhancing baseline cognitive abilities [6–9]. However, while existing literature has predominantly emphasized participation in cognitively stimulating activities, comparatively less attention has been directed toward the internal regulatory capacities that may influence how effectively individuals engage with and benefit from such activities. One such capacity is metacognition, which may represent a critical yet underexplored dynamic contributor to cognitive reserve [10].

Metacognition, refers to the ability to monitor one’s own cognitive skills (metacognitive monitoring) and to subsequently regulate and enhance one’s cognitive performance (metacognitive control) [11]. Core cognitive processes (like memory encoding or retrieval) occur at the object level, overseen by a higher meta-level, as proposed by Nelson and Narens [12]. Information flows upward via monitoring (e.g., judgments of learning or feelings of knowing) from the object level to the meta-level, while downward control (e.g., choosing an alternative problem-solving method) adjusts cognition based on those assessments [12]. Metacognitive skills are crucial for monitoring and regulating everyday activities, including communication, managing finances, driving, problem-solving, learning, decision-making, and social interactions across adulthood [13,14]. Conversely, reduced metacognitive skills, particularly evident during middle and later adulthood, are known to be associated with cognitive failure and difficulty in implementing cognitive strategies, avoidance of cognitive activity, decreased motivation, and reduced cognitive performance [15]. Metacognitive deficits have been linked to structural and functional changes in the brain, including decreased activity in the prefrontal and anterior temporal regions, atrophy in the frontal and parietal lobes, and alterations in the left angular gyrus, particularly in middle and later adulthood [16–19].

Metacognitive abilities may serve as a key mediator through which cognitive reserve exerts its positive effects on cognitive performance across the aging process [10]. Active models of cognitive reserve emphasize flexible and efficient deployment of executive processes, such as monitoring, switching, and inhibition, as key mechanisms supporting resilience to brain aging and pathology [20,21]. These processes closely align with metacognitive models that distinguish between monitoring of cognitive states and control of strategy selection and effort allocation [12]. Integrating these frameworks, metacognition can be conceptualized as a higher-order regulatory system that dynamically governs the deployment of executive resources underlying cognitive reserve. Variability in metacognitive efficiency may therefore explain individual differences in the expression of cognitive reserve beyond structural brain reserve or life-course proxies. Consistent with this view, metacognition has been shown to mediate age-related cognitive changes [10]. Such mediation seems to be possible through both the critical metacognitive components, i.e., monitoring and control. According to the monitoring-affects-control hypothesis [22], deficient metacognitive monitoring, characterized by both overestimation and underestimation of cognitive abilities, may hinder the self-regulation of cognitive strategies. Appropriate selection of adaptive strategies is crucial for protecting aging individuals against cognitive decline, as per the strategy-deficit hypothesis [23]. Therefore, underlying metacognitive impairments pose a significant threat to cognitive functionality.

Metacognitive skills change with age, especially considering the age-related atrophy of prefrontal and parietal regions [24–26]. Aspects like monitoring efficiency have shown mixed trends in existing studies with some demonstrating preserved ability with aging [27], while others suggest declines in prediction and judgment of learning, along with inappropriate overconfidence with age [28]. In contrast, metacognitive control shows consistent age-related decline, with deficient task adjustment and strategy manipulation [29]. Recent studies have found age related metacognitive differences emerging as early as middle adulthood, with middle aged adults (MAAs) exhibiting significantly lower self-efficacy as measured by estimation performance discrepancy on short term and working memory recall tasks even when recall and recognition performance remains stable [30,31]. Another study revealed a tendency among MAAs to significantly overestimate their short-term memory capabilities [25]. Moreover, a substantial proportion of MAAs demonstrate inadequate metacognitive skills, with 52% being characterized as mis-estimators of their cognitive abilities [32].

Middle age, a period between 40–65 years of age [33], offers a critical opportunity for preventive interventions before the onset of significant brain damage [34]. Strengthening cognitive functions in midlife, well before the onset of cognitive disorders, has the potential to delay symptoms, reduce their severity, and eventually prevent Alzheimer's disease later in life [34,35]. Consequently, several recent studies have reported benefits of cognitive training programs in enhancing baseline cognitive abilities of healthy aging MAAs [7–9,36–38]. A recent review and meta-analysis of cognitive training in midlife further underscored the importance of this period and highlighted the potential of such interventions in promoting cognitive and brain health [9]. These findings reinforce the idea that cognitive training may enhance compensatory processes, such as strategic regulation, monitoring, and more efficient utilization of available cognitive resources, thereby potentially supporting cognitive performance in midlife despite underlying biological changes characteristic of this age period. Along similar lines, metacognitive training programs introduced during midlife, with the explicit goal of strengthening cognitive functioning through greater awareness and regulation, represent an under researched yet potentially powerful approach to cognitive health promotion.

Metacognitive strategy training is aimed at enhancing self-awareness, self-monitoring, and the capacity to select and utilize cognitive strategies that support goal-oriented behavior such as communication [39–41]. These strategies serve as a mirror in helping one reflect on the outcome of their performance, identify their cognitive strengths and limitations, and identify any changes in their cognitive status. Metacognitive strategy training focuses on metacognitive processes, helping individuals develop self-awareness, self-monitoring, and self-regulation of their own cognitive performance [42]. For example, in the study by Bampa et al. [43] participants in the experimental group engaged in cognitive tasks such as recall, problem-solving, and attention exercises; with metacognitive component of reflection, which involved evaluating task difficulty, monitoring their own performance, and generating strategies for improvement. Angel et al. [10] proposed that focusing on metacognitive skills in training interventions could be particularly effective in optimizing cognitive performance among neurotypically aging individuals, given the crucial role of metacognitive abilities in cognitive function during aging. Given the importance of metacognitive skills, promoting training programs aimed at improving these skills seem to be a promising approach toward bolstering cognitive reserves in neurotypically aging adults.

Metacognitive strategy training has shown promise across various cognitive disorders, demonstrating effectiveness in improving cognitive and metacognitive abilities [44–47]. Research has highlighted its role in enhancing receptive skills in acquired brain injury [44], social communication skills in traumatic brain injury [46], and word retrieval skills in Aphasia [47]. The metacognitive strategy intervention, named ‘IMPACT’, tailored for cognitive communicative disorders, involving steps such as identifying a goal (I), planning (M), predicting performance (P), acting it out (A), assessing success (C), and trying again (T), has been proven effective in enhancing self-awareness in adults with acquired brain injuries [45]. Additionally, a prominent program, Cognitive Orientation to Daily Occupational Performance (CO-OP), which employs the ‘goal-plan-do-check’ strategy, improves daily life functions, cognitive performance, and quality of life in individuals with acquired cognitive communication disorders [39,41]. Another effective approach, the Multi-Context Approach, employs guided questioning or mediation, facilitates error awareness and monitoring, self-evaluation of performance, and generation of strategies among individuals with brain injury [42]. Metacognitive strategy training has demonstrated effectiveness in the context of neurodegenerative cognitive-communicative disorders [43,48]. Pikouli et al. demonstrated its role in enhancing decision-making skills in amnestic MCI, whereas Bampa et al. reported that incorporating self-testing strategies improved cognitive and metacognitive abilities in individuals with amnestic MCI, with benefits sustained six months post-intervention, suggesting potential in delaying the onset of dementia [43,48].

In the context of healthy ageing, employing metacognitive strategies to proactively manage and achieve daily goals can help offset age-related cognitive declines and optimize cognitive functioning [49–51]. On the basis of these premises, myriads of studies have incorporated metacognitive strategy training approaches in their intervention programmes with old-aged adults. For instance, the Everyday Memory and Metamemory Intervention, which includes ‘stop-think-plan-act’ metacognitive strategy training, was effective in enhancing memory skills among older adults [50]. Additionally, Bailey et al. reported that a self-testing metacognitive strategy was effective in improving learning in older adults [51]. A recent scoping review of metacognitive training programs among neurotypically aging populations and individuals with cognitive-communication disorders found that most existing programs have either been designed for clinical populations or evaluated primarily for their efficacy in older adults [49]. Emphasizing the preventive potential of metacognitive training in promoting cognitive well-being among neurotypical adults, the review advocated for its early introduction during midlife [49].

### The present study

Given the importance of metacognitive ability in identifying early cognitive impairments [52], its mediating effect on enhancing cognitive reserves with aging [10], the documented prevalence of poor metacognitive skills among individuals in middle adulthood [24,25,30], and the potential significance of metacognitive strategy training programs for this age group, there is a need to explore the impact of metacognitive strategy training among MAAs. However, before undertaking such investigations, a crucial preliminary step involves the systematic and scientific development of a metacognitive strategy training program specifically designed for this demographic, along with their feedback on its design, implementation, and impact. This step is essential before any large-scale research or clinical rollout can be performed. Thus, the present study aimed to address this gap by developing a comprehensive metacognitive strategy training program tailored specifically for neurotypical MAAs, along with an initial feasibility assessment to inform its future utility.

The present study followed the National Institutes of Health (NIH) stage model for behavioral intervention development [53]. The model offers a direction for developing highly effective behavioral interventions that could be easily implemented to increase health and well-being. The Stage Model includes six stages, i.e., basic science (Stage 0), intervention generation, refinement, modification, and adaptation (Stage IA) and pilot testing (Stage IB); traditional efficacy testing (Stage II); efficacy testing with real-world providers (Stage III); effectiveness research (Stage IV) and; dissemination and implementation research (Stage V). The present study aligns with Stages IA and IB, i.e., intervention development and feasibility testing of the MCST protocol. The study was conducted in two parts: Study 1 focused on Stage IA, involving the generation or adaptation or modification of the MCST intervention, whereas Study 2 addressed Stage IB, which entailed field testing of the MCST program with MAAs. The following hypotheses were tested in the feasibility phase of the present study, on the basis of the framework of the National Center for Complementary and Integrative Health (NCCIH), which provides guidance for the design and conduct of pilot studies:

- At least 90% of the recruited MAAs would be retained throughout the MCST program.
- At least 80% of the MAAs would perceive the MCST program as engaging and acceptable.
- The participants of the experimental group would demonstrate significantly greater improvements in cognitive and metacognitive aspects of the trained tasks compared to the control group, following the MCST.

Cognitive reserve is a broad construct, encompassing factors such as education, occupation, engagement in physically and cognitively stimulating activities. In the present study, we evaluated the preliminary effects of the MCST on cognitive and metacognitive performance using the CMT recall task. While cognitive reserve itself was not directly measured, any positive trends in cognitive performance on this task were considered as one of the preliminary indicators of cognitive reserve, given that research suggests a bidirectional relationship between cognitive functioning and cognitive reserve [54].

The methodologies and findings of these two studies are presented independently, followed by an integrated discussion. The present study is reported in accordance with the Template for Intervention Description and Replication (TIDieR) guidelines [55],which are recommended for reporting intervention development studies, including nonrandomized feasibility study.

## Study 1

### Materials and methods

The first study focused on the development of an MCST protocol to foster optimum metacognitive skills among MAAs using a consensus-building method known as the Nominal Group Technique (NGT). The study was conducted between April 2023 and February 2024. The approval was obtained from the institutional ethics committee before the commencement of the study (IEC KMC MLR 03/2023/116).

#### Nominal group technique.

NGT is a methodical process that involves taking several stages to reach consensus on ideas or viewpoints among a small group of people ensuring that everyone has a chance to represent their ideas [56]. A typical NGT includes four steps, including idea generation, round-robin, group discussion, and ranking. Modified Nominal Group technique (mNGT) is the adapted method of a typical NGT, where certain steps are modified by either adding or omitting them depending on the research study. In the present study, we conducted two rounds of mNGT. The first round of mNGT was carried out to select a suitable metacognitive strategy that could foster optimal metacognitive skills among MAAs. The second round of mNGT was conducted to identify the execution-related aspects of the MCST protocol. Each round of mNGT was conducted in a face-to-face structured group discussion format, lasting for approximately 60 minutes each.

**mNGT Panel.** Recognizing the potential role of SLPs in metacognitive strategy training, and their established involvement in the development, implementation, and evaluation of such interventions for cognitive communication disorders [44,46,47,49,57], the present study included five SLPs (Mean age: 40.6 + 12.4 years; Male −1, Female −4) serving as healthcare professionals in the mNGT panel. Each of them had over 3 years of experience in the field of cognitive communicative sciences and had clinical experience working with aging adults. All the panelists had either completed their doctoral degree and held academic positions (n = 3) or were pursuing (n = 2) it in cognitive-communication sciences. The primary researcher in the study served as the facilitator for the mNGT, consistent with other mNGT studies [58,59]. The facilitator’s role was limited to presenting each strategies and manging the procedural steps of the mNGT. All evaluative decisions, including judgements of redundancy, were made independently by the panel members during subsequent rounds. The participants were recruited through purposive sampling. Written informed consent was obtained from all participants.

**mNGT- Round 1.** The first round of the mNGT aimed to select an appropriate metacognitive strategy that could facilitate optimum metacognitive skills among MAAs. To select a metacognitive strategy, we followed the three steps of the mNGT i.e. orientation of various metacognitive strategies, clarification among the panel members about the shortlisted strategies, and ranking of the most suitable metacognitive strategy. In the first step, *orientation*, the panel members were explained about the purpose of the meeting and were presented with the list of available metacognitive strategies. Based on the literature review, the research team compiled a list of commonly reported metacognitive strategies [39,41,45,46,50,60,61]. All participants were explained that metacognitive strategies are techniques that assist individuals in monitoring their own cognitive performance and progress, in contrast to cognitive strategies that aid in enhancing cognitive performance. Each strategy was described based on its parent source, as proposed by the original authors, and was explained by the facilitator with examples and clarifications as needed. This approach was intended to preserve the authenticity of the original strategies and minimize interpretive bias. The facilitator ensured that all the participants had a complete understanding of each of the strategies presented by asking the panelists to provide a summary. In the following step, *clarification*, the group discussed their views and opinions about the relevance of each metacognitive strategy in fostering metacognitive skills. Panel members could exclude, include, or modify strategies in this round. Any changes arising through discussions were recorded by the facilitator. In the final step, *ranking*, each panel member was asked to independently rank each strategy obtained from the previous step for its relevance. The average rank obtained for each metacognitive strategy was calculated. The outcome of the ranking was recorded, and the highest-rated metacognitive strategy (highest average ranking) was considered.

**mNGT- Round 2**. Following the selection of a suitable metacognitive strategy, the second round of the mNGT was conducted with the aim of identifying the execution-related aspects of the MCST protocol. The steps included in this round were silent generation, round robin, clarification, and ranking. At the outset, all the panel members were explained that the main objective of this mNGT was to determine the number of sessions, the process for selecting goals, and methods for tracking the progress of strategy usage in MCST. In the first step, *silent generation*, all participants individually jotted down their suggestions on the number of sessions and ideas pertaining to goal selection and methods for tracking progress. A maximum of 20 mins was provided to generate ideas. Following this, the *round-robin* step was carried out where participants read aloud their ideas, and the facilitator recorded them on the white board. In the subsequent step, *clarification*, the panel members engaged in an open discussion on each of the suggestions, with the opportunity to add, modify, and combine their ideas. This clarification enabled informed decision-making during the ranking process. Subsequently, the *ranking* of the most suitable goals selected to execute the MCST protocol, the number of sessions, and methods to track the responses were done by the panel members and the highest-ranked aspects were considered for the execution process.

## Results

### The MCST protocol

To develop the MCST protocol, two rounds of mNGT were employed. The first round of the mNGT focused on selecting an appropriate metacognitive strategy whereas the second round of the mNGT focused on determining the execution related aspects of the MCST.

#### Strategy related aspects of MCST.

To derive the specific strategy for the MCST protocol, a three-step mNGT process (orientation, clarification, and ranking) was employed. The list of metacognitive strategies that were provided to the panelists during the orientation phase is depicted in Table 1. During the clarification step, certain metacognitive strategies, such as “feedback, self-prediction, self-reflection, and self-monitoring,” as well as “problem-solving training,” were removed from the list, as these strategies were found to be redundant by the panel members. Additionally, when different strategies were reviewed during the clarification stage, the discussion among panel members resulted in the adoption of a metacognitive strategy that combines the salient elements of each of the shortlisted strategies and offers a comprehensive framework. This adapted strategy comprises elements pertaining to pausing before a desired task, pondering over its different aspects, strategizing a plan for successful execution, actual performance in the task, and post-execution reflection was termed as ‘stop-think-plan-do-check’. All the shortlisted strategies were ranked during the third step of the mNGT and the ‘stop-think-plan-do-check’ strategy was found to receive the highest ranking by the panel members. The results of each step of the first round of the mNGT are depicted in Table 1. A detailed description of the steps of this adapted strategy, as jointly discussed by the panel members, is outlined in Table 2.

**Table 1:** Results from the first round of Nominal Group Technique (mNGT).

Metacognitive strategies discussed during orientation (step 1)	Metacognitive strategies shortlisted during the clarification stage (step 2)	Metacognitive strategies as per ranking (step3)
• Identifying the goal; anticipate reaching the gaol; identify possible solutions to challenges; self-monitor and evaluate progress; modify strategy usage if required [44] • Feedback; self-prediction; self-reflection and self-monitoring; modelling [46] • Stop – think – plan – act [50] • Guided questioning: identify challenges and self-generate strategies, self-assessment of success or challenge, strategies used, and strategies to be modified [60] • Self-check lists [60] • Goal – plan – do-check [41] • Self-prediction before performance; self-evaluation following performance [61] • WSTC – What should I be doing? Select a strategy. Try the strategy. Check the strategy [39] • PST – problem-solving training that includes problem orientation, problem definition, generative alternatives, decision-making, and solution verification [39] • GMT (goal management training) – Stop. Define main task. List steps. Learn steps. Execute task. Check [39]	• Identifying the goal; anticipate reaching the goal; identify possible solutions to challenges; self-monitor and evaluate progress; modify strategy usage if required • Self-prediction before performance; self-evaluation following performance • Stop-Think-Plan-Act • Goal – plan – do-check • Stop-Think-Plan-Do-Check • WSTC – What should I be doing? Select a strategy. Try the strategy. Check the strategy • GMT (goal management training) – Stop. Define main task. List steps. Learn steps. Execute task. Check • Self-checklists • Guided questioning	1. Stop-Think-Plan-Do-Check 2. GMT (goal management training) – Stop. Define main task. List steps. Learn steps. Execute task. Check 3. Identifying the goal; anticipate reaching the goal; identify possible solutions to challenges; self-monitor and evaluate progress; modify strategy usage if required 4. Goal – plan – do-check 5. Stop-Think-Plan-Act 6. WSTC – What should I be doing? Select a strategy. Try the strategy. Check the strategy 7. Self-checklists 8. Guided questioning 9. Self-prediction before performance; self-evaluation following performance

**Table 2:** Description of the adapted metacognitive strategy.

**The ‘Stop-Think-Plan-Do-Check’ strategy** The first step, *stop*, focuses on taking a pause before starting any activity and think about it. The following step, *think,* encourages participants to think about the nature and demands of their activity along with an estimation of their ability to perform it. The ‘*plan’* step encourages participants to think of efficient strategies and methods that can aid them in successfully completing their desired task. In the subsequent step, *do*, participants are asked to complete their desired task by adopting the strategies planned. The last step, *check’* included reflecting on how well the task was completed and if it matched their predicted estimation. Checking also aids in determining if the strategies adopted to complete the task was successful or not and if any modifications need to be made to the plan. This 5-step strategy guides an individual in self-reflecting and self-monitoring their cognitive performance.

#### Execution-related aspects of MCST.

The second round of the mNGT aimed at identifying the execution-related aspects of the MCST protocol such as determining the duration of training, methods to identify goals to practice the STPDC, and methods to track the progress of the training. The second round of the mNGT included four steps namely, the silent generation of ideas, round robin, clarification, and ranking. The results of each step are provided in Table 3. The first two steps resulted in the generation of three training durations. In the clarification phase, it was narrowed down to three options, i.e., four, six, or seven sessions, on the premise that MAAs would adhere to shorter training programs because of their hectic schedules. The final step of the ranking resulted in the highest ranking for the seven-session protocol (2 testing sessions and 5 training sessions). With respect to methods for selecting goals and tracking progress, four ideas were listed for each through the first two steps of mNGT. No revisions were made to this list during the clarification step. The ranking process resulted in highest ranking for ‘client-centred personalized goals’ for goal selection and ‘daily dairy’ to track progress.

**Table 3:** Results of second round modified Nominal Group Technique (mNGT).

Execution-related aspects of the MCST program	Execution-related aspects identified from silent generation and round robin (step1 and 2)	Execution-related aspects identified through clarification (step3)	Ranking of the execution-related aspects (step4)
**Training duration**	• 7 sessions • 4-6 sessions • 10 sessions	• 4 sessions • 6 sessions • 7 sessions	1. 7 sessions 2. 6 sessions 3. 4 sessions
**Selection of goals to practice STPDC**	• Pre-designed goal bank • Goals identified from Metacognitive Knowledge in Everyday memory, attention, executive function (MKEA/M/EF) questionnaire [62] • Client-centred personalized goals [63,64] • Implementing a uniform cognitive communicative goal across all participants	• Pre-designed goal bank • Goals identified from MKEs questionnaire • Client-centred personalized goals • Implementing a uniform cognitive communicative goal across all participants	1. Client-centred personalized goals 2. Pre-designed goal bank 3. Goals identified from MKEs questionnaire 4. Implementing a uniform cognitive communicative goal across all participants
**Methods to track the progress of MCST**	• Semi-structured interview • Goal Attainment Scaling (GAS) [46] • Daily diary method • Goal performance and satisfaction rating scale	• Semi-structured interview • Goal Attainment Scaling (GAS) • Daily diary method • Goal performance and satisfaction scale	1. Daily diary method 2. Goal Attainment Scaling (GAS) 3. Semi-structured interview 4. Goal performance and satisfaction scale

**Client-centered personalized goals.** The client-centered approach [63] was used to identify goals for practicing the STPDC metacognitive strategy. As part of this goal selection approach, the participants identify goals from their daily life that were relevant and motivating for them to improve upon. The participants were encouraged to think of a cognitive goal which was part of their routine, and they wished to improve upon. The participant, along with the researcher, defined a specific goal. In case of any difficulty, the researcher provided with examples to facilitate the selection of an appropriate goal. The participants were subsequently explained how the STPDC could be applied to their personal goals. Table 4 exemplifies the integration of metacognitive strategies into everyday activities, providing an illustration of their application.

**Table 4:** Integration of metacognitive strategy in everyday activities.

**Integration of metacognitive strategy in everyday activities** *Client centered personalized goal* To recall a list of 20 items to purchase from the supermarket without using a list. *Integration of the metacognitive strategy* Stop: Before you go to the grocery store to purchase your items, take a pause to think about your goal. Think: Think about how many of the 20 items you think you will be able to recall (Ex: I think I will be able to recall only 13 of the 20 items without relying on the list). Plan: Includes thinking of methods that could help to effectively recall all 20 items. For instance, one may benefit from grouping purchase items into smaller categories making them easier to recollect. Do: Shopping at the supermarket without using any written list and using the chunking strategy to recollect purchase items Check: After completing the supermarket shopping, individuals assess whether they purchased all 20 items and compare their performance to their initial estimation (13 items). This evaluation can include noting whether they recollected all 20 items, recollected more than 13 items, or recollected fewer than 13 items. Following this, individuals assess if chunking strategy helped in recollecting purchase items and if more strategies must be used for enhancing memory retention during future shopping endeavors.

**Daily diary.** The objective of the daily diary was to document the progress of MCST sessions. The daily diary was used to document goals identified by the participants, their proficiency in executing the STPDC, the perceived benefits of the metacognitive strategy, and the generalizability of the metacognitive strategy usage (if any), and to obtain feedback on the client’s experience of strategy usage. The daily diary was filled out at the end of each session by the participant via a Google form provided by the researcher. The details of each of these measures captured via Google form are provided in Table 5.

**Table 5:** Format of the daily diary.

Construct	Question	Response format
Details on personal goals	Which *daily task* are you working on to improve your understanding about your cognitive (or brain) abilities?	Open-ended
	Did you get a chance to perform your daily task?	Yes / No
Proficiency in executing each step of the metacognitive strategy	1. Before beginning the activity/ task how well were you able to *take a pause* to think about the task 2. Before beginning the activity/ task, how well were you able to *think* about the nature and demands about the task along with my potential/ capabilities to perform it. 3. Before beginning the activity/ task, how well were you *able to plan* a strategy to successfully accomplish the task while keeping in mind the task demands and my capacity. 4. How well were you able to *perform* the task? 5. After completing the task/ activity, how well were you able to *check* if the task went as per the plan and reflect on the facilitators/barriers to ensure better performance next time	Rating scale (1-not at all to 10 – very well)
Perception about the benefits of metacognitive strategy	Did you find the ‘*stop-think-plan-do-check’* strategy was beneficial in understanding your ability to perform the task?	Yes/No
Usage of strategy on other goals	Did you apply the ‘*stop-think-plan-do-check’* strategy on any other task apart from the one that was discussed? If yes, which activity? And was it helpful?	Open-ended response
General feedback about to strategy	Your final thoughts about using ‘*stop-think-plan-do-check’* strategy?	Open-ended response

**Fig 1:**
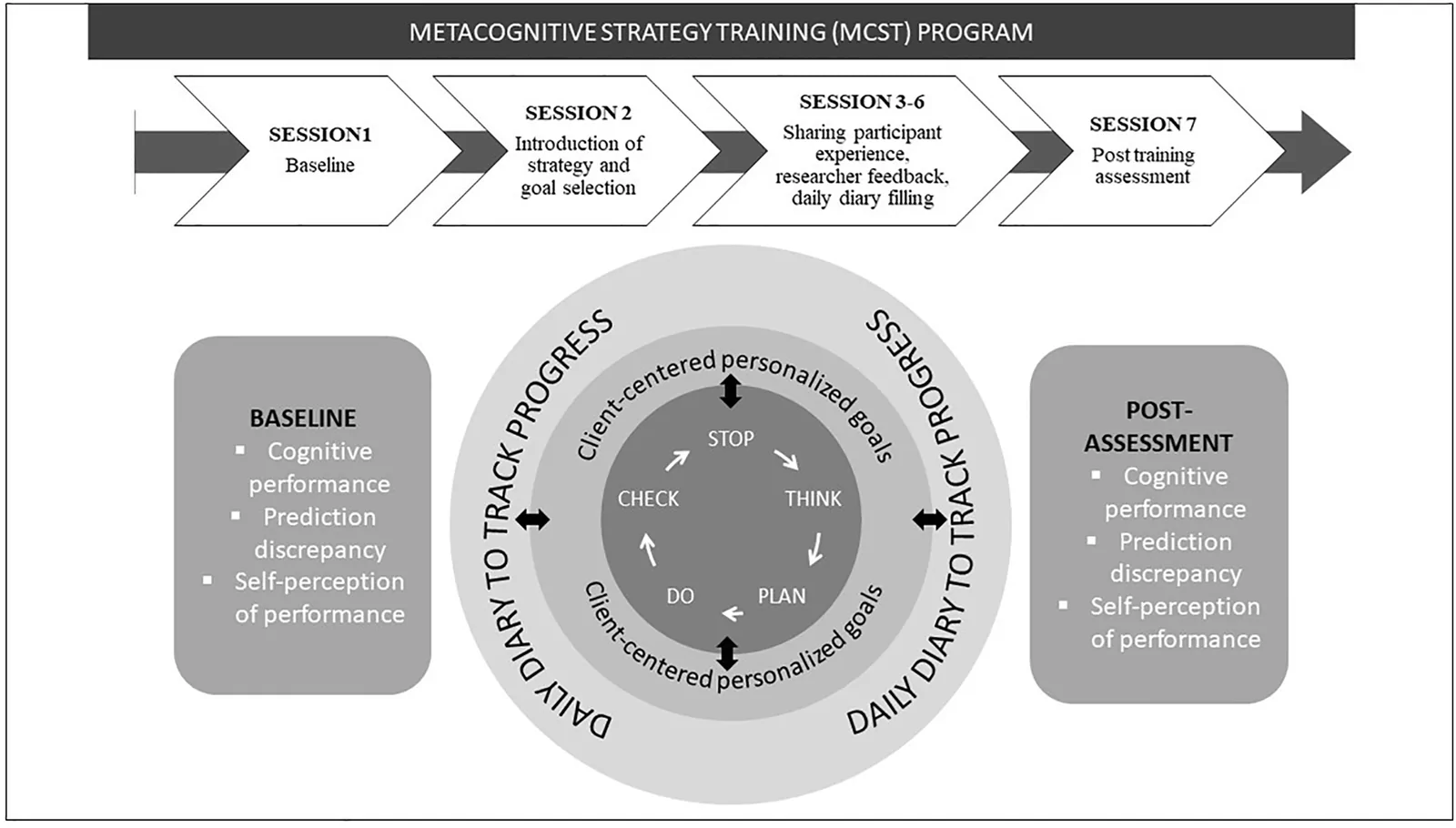
Key elements of the Metacognitive Strategy Training (MCST) protocol.

The MCST protocol was devised based on the findings obtained from two rounds of the mNGT. The critical elements of the MCST protocol along with the salient aspects for each of the seven sessions are outlined in Fig 1.

## Study 2

### Materials and methods

The second study focused on feasibility testing of the developed MCST protocol with twenty healthy MAAs, who were assigned to Control Group (CG) or Experimental Group (EG) based on their willingness. A non-randomized controlled experiment was conducted to evaluate the protocol adherence, engagement, acceptability, and determine whether it led to desirable cognitive and metacognitive changes. Such non-randomized feasibility studies are typically conducted at an earlier stage of intervention development before conducting randomized trials [65]. The study was conducted between April 2023 and February 2024. The approval was obtained from the institutional ethics committee before the commencement of the study (IEC KMC MLR 03/2023/116).

#### Participants.

In accordance with the recommendations of Moore et al. [66], who suggested a minimum of 12 participants for pilot and feasibility studies to establish preliminary estimates of averages and variability for future larger trials, our study recruited 20 participants with 10 participants in EG and CG, respectively. This sample size was deemed suitable and practical for an early-phase feasibility investigation, facilitating the generation of significant preliminary data. Neurotypical MAAs between 40 and 65 years of age (mean age: 50.2 + 5.3 years) [33] with a minimum of 14 years of educational qualification from an English medium institution, proficiency in English as ascertained through the Learning Experience and Proficiency Questionnaire [67], belonging to middle socioeconomic status [68], and having average general score below 0.49 (indicating no disability) on the World Health Organization Disability Assessment Schedule (WHODAS 2.0) [69] were included. Participants scoring below 26 on the Mini Mental State Examination (MMSE) [70] and having any history of neurological or psychological disorders were excluded from the study. All participants included in the study were employed. Participants were recruited from the community via convenience sampling using a public advertisement that provided neutral information about the structure and logistics of the program (number of sessions, location, and general focus in cognitive activities), without making claims about benefits. Based on this information, participants self-selected into CG or EG according to their willingness to participate in the training program. Written informed consent was obtained from all participants prior to enrollment, with explicit clarification that participation was entirely voluntary and that they were free to withdraw at any stage without consequence. This procedure ensured that participants were not subjected to any undue pressure or obligation to continue or to perform in a particular manner. No incentives or compensation were provided to the participants in this study. The recruitment period of the study spanned from 1^st^ October, 2023–31^st^ January, 2024.

#### Outcome measures.

**Participant recruitment, retention, and adherence.** The recruitment rate was defined as the proportion of participants who consented to enroll in the study. The retention rate reflects the proportion of participants who completed the full protocol, and the adherence rate captures the extent to which participants attended and completed all intervention sessions.

**Participant engagement in intervention.** The reflections from the daily diary were used to track progress across the sessions and were therefore filled only by the EG. The format of the daily diary, as finalized in Study 1, is provided in Table 5. The results from the daily diary are described descriptively in terms of goal selection, the benefits and generalizability of strategy usage, and the ratings obtained regarding self-perceived proficiency with the taught strategy.

**Intervention acceptability.** A custom-made feasibility questionnaire (Table 7) was used to gather feedback from the EG participants on the MCST program, evaluating parameters such as the comprehensibility of program instructions, content delivery, engagement levels, acceptability, time commitment, examiner support throughout the training, fatigue, anxiety, relatability, logistic considerations, and overall training experience. The participants rated each parameter on a 5-point rating scale (5: Strongly agree to 1: strongly disagree). This questionnaire was content-validated by six speech-language pathologists to evaluate the appropriateness and relevance of its items. It achieved a content validity index (CVI) of 0.83 [71], indicating that the tool is suitable for assessing participants’ acceptability of the intervention.

**Cognitive and metacognitive aspects related to Contextual memory test (CMT)** [72]**.** CMT assesses memory and metamemory capacity related to everyday contexts. The CMT domains include self-efficacy, self-perception, and evaluation of performance, immediate recall, delayed recall, total recall, total strategy use, and general self-awareness. The CMT is reported to have good construct and discriminant validity, sufficient test-retest reliability and acceptable minimal detectable change [73]. In the present study, only the prediction of performance and immediate recall was used to assess the outcome measure.

***Cognitive performance: Immediate recall.*** The immediate recall of CMT was used to assess cognitive performance. The task involved displaying a set of 20 pictures from a specific context to the participants for 1.5 minutes, followed by an immediate recall of as many items as possible. The number of items recalled was calculated. The immediate recall scores were calculated for both groups (EG and CG) at baseline and post-training assessment.

***Absolute self-estimation.*** The absolute self-estimation was assessed using the prediction discrepancy of the CMT task. Prior to the administration of the task, the participants were asked to predict the number of items they thought they would recall in the CMT task. The difference between the predicted value and the performance score on the CMT was considered as the prediction discrepancy. The absolute self-estimation was calculated for both groups (EG and CG) at baseline and post-training assessment.

***Self-perception of performance*.** To assess self-perception of task performance, the Dynamic Test After-Task Awareness Interview of Multi-Context Approach [74] was used. The interviews revealed participants self-awareness of their performance through a series of questions that gradually became more specific. Considering that the present program was designed for neurotypicals, to document even the subtle changes in awareness, we modified the interview questions and ratings as outlined in the S1 Appendix. The responses were rated on a 7-point rating scale ranging from 1 (good awareness) to 7 (unaware). Since self-perception of performance involves mediation questions that could serve as a feedback mechanism, it was administered only to the EG to prevent unintended intervention effects in the CG.

#### Procedure.

The feasibility testing comprised of seven daily individualized sessions (five training sessions and two assessment sessions) conducted in a well-lit, distraction free room within the participant’s home environment. The participants in the Experimental Group (EG) completed all seven sessions, whereas those in the Control Group (CG) participated only in the two assessment sessions (Session 1 and Session 7). The CG did not receive any form of training between the two assessment sessions.

The first session focused on obtaining the baseline of outcome measures using the CMT, which was completed by both the CG and the EG. The participants were provided with instructions about the task. Once comprehension of instruction was ascertained, prediction ratings were obtained for the CMT task. Following this, the task was administered to assess the immediate recall abilities. After the administration of the task, self-perception of performance was assessed (only for the EG) by using the adapted version of the Dynamic Test After-Task Awareness Interview. The baseline assessment took approximately 15 minutes.

Only the EG participated in sessions 2–6. The second session focused on introducing the metacognitive strategy to the participants. The steps of the STPDC strategy were displayed on a laptop screen using presentation slides, accompanied by verbal directions to facilitate participant’s comprehension. To verify that participants successfully understood the strategy, they were asked to explain it back to the researcher. In case of any confusion, the researcher provided additional explanations to clarify the strategy. The participants were subsequently encouraged to select a personal cognitive goal (client specific) relevant to their daily routine where they could practice the STPDC strategy. Sufficient examples were provided to the participants, who were encouraged to state their goals in a similar manner. The participants were encouraged to define specific goals as elaborated in the results section of Study 1. Sessions 3–6 followed similar structure. The sessions began by asking the participants to describe their experience of strategy usage. The researcher provided feedback to the participants based on the description of their experience. If participants were found to be applying the strategy effectively, their application was reinforced to encourage continuation. When any drawbacks were observed in strategy usage, corrective feedback was provided to address the challenges. The researcher provided suggestions on how to improve the implementation of the strategy and execute each step more proficiently while also encouraging participants to try using the strategy for other activities and tasks in their daily life. The sessions ended with participants completing their daily diary entry. Each training session lasted for approximately 20 minutes.

Session 7 included conducting a reassessment of all the baseline measures (similar to session 1) on both the EG and CG. In addition, a feasibility questionnaire was administered to the EG to obtain feedback regarding the MCST training.

#### Data analysis.

To evaluate the feasibility of the MCST protocol, the daily diary method was employed to monitor session-wise progress and participant engagement with the intervention protocol, and the responses were descriptively reported for each participant. This method made it possible to thoroughly examine participant participation, adherence, and experiences with the MCST protocol, offering insightful information on its applicability and potential for implementation. The self-perceived proficiency of metacognitive strategy usage, as obtained from daily diary, was subjected to descriptive statistics, and session-wise mean and standard deviations of the proficiency ratings were estimated. To examine the effects of the MCST on cognitive performance and absolute self-estimation, Quade non-parametric analysis of covariance was used. The effect size for these measures was assessed using Cliffs delta. To examine the effect on self-perception of performance, the Wilcoxin signed-rank test was used. A p-value of 0.05 was considered for significance. Given the feasibility and exploratory nature of the study, no adjustments for multiple comparisons were applied. Accordingly, p-values were interpreted descriptively within the exploratory context of the study. The responses from the feasibility questionnaire were descriptively analyzed via frequency measures and Cronbach’s alpha was calculated to check for its internal consistency.

## Results

### Participant recruitment, retention, and adherence

The developed MCST program was tested on twenty MAAs. The participants in both groups were significantly similar on demographic variables such as age (*t*(18)=0.29, *p* = 0.77), gender (χ² [1,20] = 0.22, *p = 0.63)*, and education (*t*(18)=1.08, *p* = 0.295) (Table 6). The study achieved full recruitment, with all 20 participants enrolling and completing the study, resulting in a recruitment and retention rate of 100%. Furthermore, all the EG participants attended and completed every training session, indicating an adherence rate of 100%.

**Table 6:** Demographic characteristics of participants by group.

	Experimental group	Control group
**Age (years)**	54.1 (6.04)	53.3 (6.11)
**Gender (%)**	Male: 40% Female: 60%	Male: 30% Female: 70%
**Education (years)**	15.1 (1.1)	14.6 (0.96)

### Participant engagement in intervention

**Fig 2:**
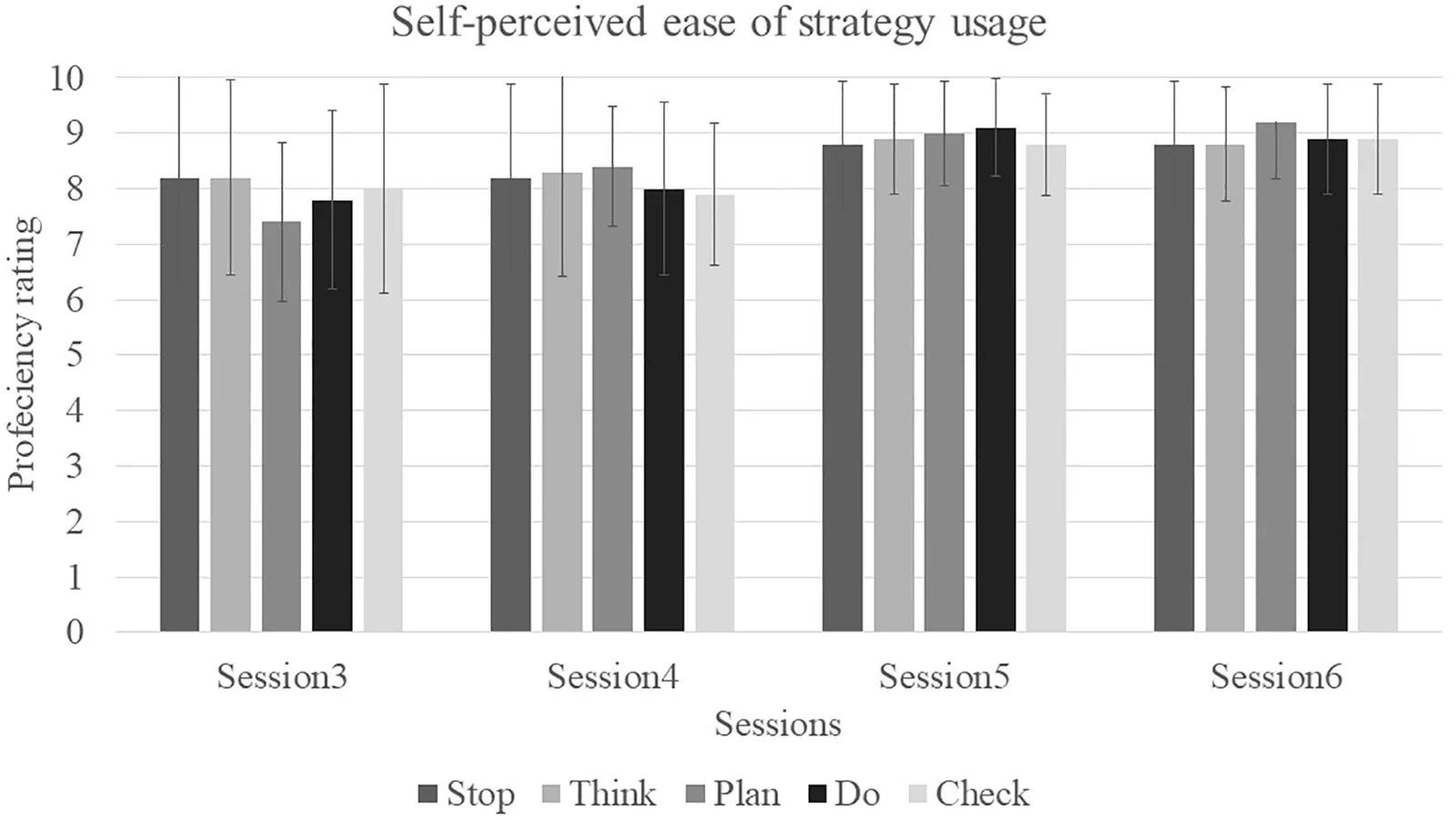
Rating on proficiency of metacognitive strategy usage.

The daily progress across sessions was tracked using a daily diary method that captured results pertaining to goals selected by each participant, proficiency of metacognitive strategy usage, self-perceived benefits of the strategy, generalization of strategy usage, and general feedback about the strategy. The results of all these measures for each participant are depicted in Table 7. All the participants were able to identify a goal to work on and found the strategy beneficial in helping them understand their cognitive abilities. Six of the 10 participants generalized the use of the metacognitive strategy to goals apart from the one that was discussed in the training sessions. The participants described the STPDC as helpful in enhancing their cognitive performance, self-efficacy, self-monitoring and self-awareness, successfully conducting their tasks, and understanding the barriers to and facilitators of their task performance. With respect to proficiency in the use of metacognitive strategies (depicted in Fig. 2), the ratings for the execution of each step in the strategy increased across sessions. The rating for ‘stop’ and ‘think’ steps increased by half a level, the ‘plan’ step increased by approximately 2 levels, and the ‘do’ and ‘check’ steps increased by almost one level on the proficiency rating scale (on the 1–10 rating scale).

**Table 7:** Daily diary results of each participant.

Participant	Goals selected	Self-perceived benefits of strategy training	Generalization of strategy usage on other daily life goals	Feedback about metacognitive strategy
P1	To assess and improve my reading attention span	Yes	• Planning of my diet meal	“It is very helpful and easy to follow. It has become my mantra”
P2	To assess and improve my ability to remember my daily tasks and appointments without relying on my calendar	Yes	• To plan my lecture classes • Trying to remember my grocery list without a list • To plan and remember my speech	“I wish I had known about this much much earlier! This strategy has facilitated a memory boost. It's a good feeling! It's beneficial, I feel I can remember rather than thinking my memory lapses are age related. A huge morale booster!”
P3	To assess my ability to remember my daily tasks and improve on it to withdraw reliance on dairy.	Yes	Nil	“Strategy has helped me. Need to work more on strategy planning. I could be able to remember more items every time”
P4	To improve my reading speed and attention span.	Yes	• To plan my lectures in class • To plan my meal preparation • To improve face-name association of students in school	“It helped me to reach the goal in the particular time. The planning was very good. It took me to a better level in my teaching strategy. I can use the strategies on all the field. Especially as a teacher, it helping me a lot. Can use this in my daily life also. Helping me to check how I am able to do my task - whether I'm doing it well or not.”
P5	To improve face name association of students in my class	Yes	• Planning daily routines without any aid • Planning my teaching lessons	“It helped me to understand my abilities. It helped me in concentrating and recalling the subject. It also helped me to evaluate my performance effectively. I was able to perform better.”
P6	To remember to carry out my tasks through the day without reliance on external aids	No (Session 1) Yes (Session 2–4)	Nil	“I think I need practice to use more effectively. It is helpful in mentally organizing my routine and in understanding my capabilities. Helpful in completing my daily routine more effectively.”
P7	To get back to the habit of reading book and improve my attention span	Yes	Nil	“It helped me start what I have been wishing to start for a long time and it is helping me to understand my abilities better. It was helpful”
P8	Remembering the key points of book chapters I read	Yes	• Completing my daily routines	“This strategy is giving more confidence about my cognitive ability because it helps me to understand my ability.”
P9	Memorizing bible verses	Yes	Nil	“It helps me plan and memorize things more effectively. I am more confident that I can memorize now. It helps me understand how I perform.”
P10	Learning a new language	Yes	• Remembering my grocery items without reliance on to-do list • Face-name association of my clients	“It made me realize that certain tasks I am really good at. Certain tasks I struggle with. But I am able to reflect why is it I’m struggling. For instance, in learning this new language, I realized I am not spending enough time on rehearsal and practice that’s why I’m taking a long time to learn it. With remembering to-do list and face recognition of my clients, I realized I am able to remember things better than I had thought for myself.”

### Intervention acceptability

The feedback obtained from the feasibility questionnaire on the design and implementation of the MCST program indicated positive responses from the participants. The majority found the program instructions easy to follow, the content effectively delivered, and the training engaging. The participants reported feeling comfortable and were willing to continue their involvement, reflecting high acceptability. They expressed satisfaction with the examiner support provided, experienced minimal fatigue, and found the training related to their experiences. The logistical aspects of the program were deemed appropriate, further facilitating participation. Overall, all the participants reported a positive experience with the MCST program. The frequency of participants responses across these parameters is depicted in Table 8. The feasibility questionnaire demonstrated acceptable internal consistency, with a Cronbach’s alpha of α = 0.767, indicating that the items reliably measured intervention acceptability

**Table 8:** Frequency of participant ratings across parameters in the feasibility questionnaire.

Statements	Strongly agree	Agree	Neutral	Disagree	Strongly disagree
Comprehensibility of instructions: • The instructions were clear and easy to follow.	80% (N = 8)	20% (N = 2)	0	0	0
Content delivery: • The training content was presented clearly and effectively.	70% (N = 7)	30% (N = 3)	0	0	0
Engagement levels: • The training sessions were engaging.	90% (N = 9)	10% (N = 1)	0	0	0
Acceptability: • I am willing to participate in the training and feel comfortable with its structure and implementation.	80% (N = 8)	20% (N = 2)	0	0	0
Time Commitment: • I found the time required for the training sessions to be manageable.	60% (N = 6)	40% (N = 4)	0	0	0
Examiner support throughout training: • The examiner offered sufficient support throughout the training.	100% (N = 10)	0	0	0	0
Fatigue: • I experienced minimal fatigue during the training sessions.	100% (N = 10)	0	0	0	0
Anxiety level: • I felt at ease during the training sessions.	100% (N = 10)	0	0	0	0
Relatability: • I found the training content relatable to my everyday context.	90% (N = 9)	10% (N = 1)	0	0	0
Logistic considerations: • Session duration: The duration of each training session was appropriate. • No. of session: The number of training sessions was sufficient. • Inter-session duration: The duration between training sessions was appropriate.	100% (N = 10) 100% (N = 10) 100% (N = 10)	0 0 0	0 0 0	0 0 0	0 0 0
Overall training experience: • Overall, my experience with the training was positive.	80% (N = 8)	20% (N = 2)	0	0	0

### Effect on cognitive performance, absolute self-estimation, and self-perception of performance

**Fig 3:**
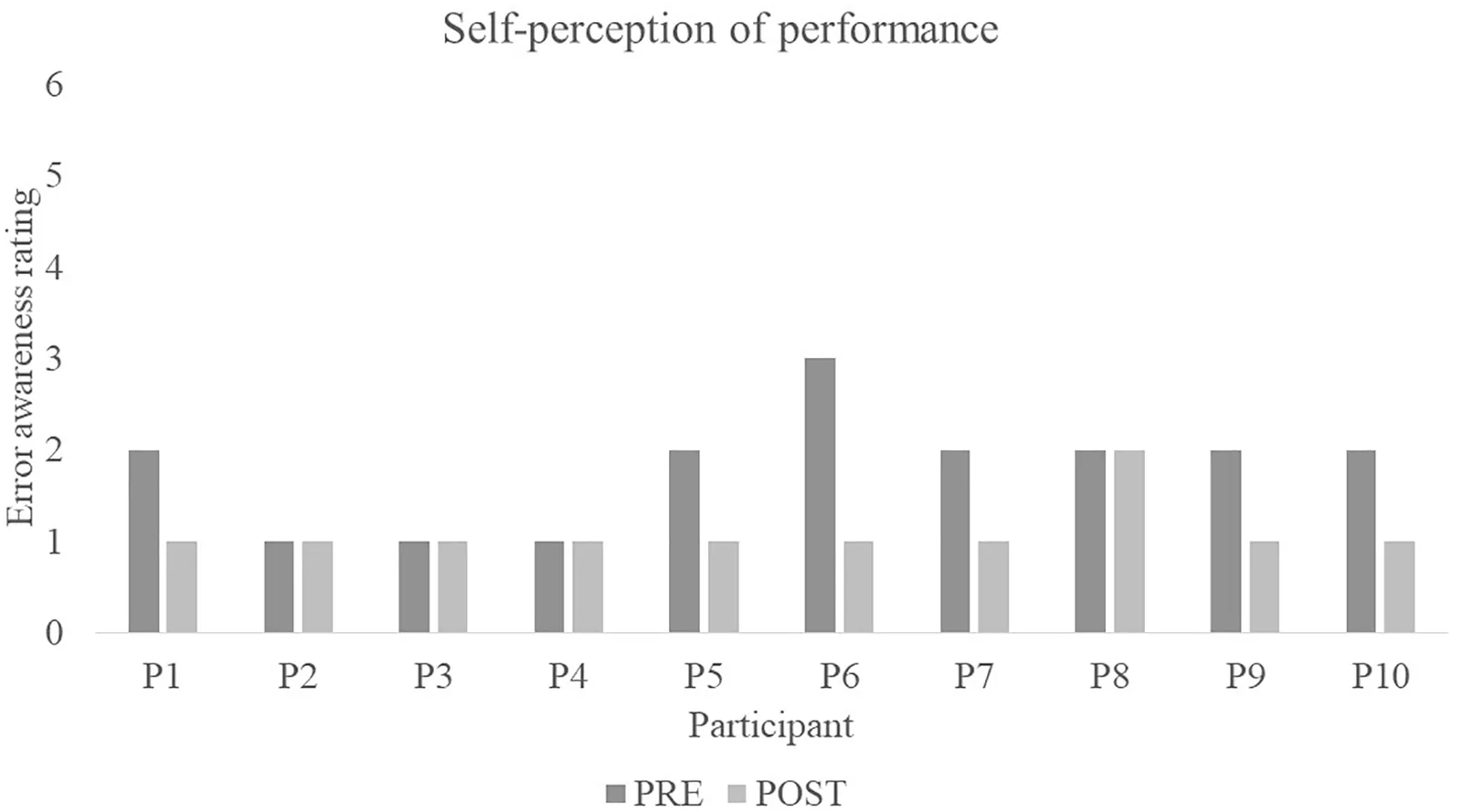
Pre and post-training self-perception of performance ratings of EG participants on CMT.

The feasibility of the training program was assessed on cognitive performance, absolute self-estimation using prediction discrepancy, and self-perception of performance using the CMT. The descriptive statistics of absolute self-estimation measure and cognitive performance is provided in Table 9. The Quade nonparametric analysis of covariance revealed a significant increase in both of these measures (ASE: Q (1,18) = 5.009, *p =* 0.038; recall: Q (1, 18) = 13.615, *p =* 0.02) for the EG compared with the CG at the post-training assessment after controlling for baseline performance (Table 9). With respect to self-perception of performance ratings, the EG demonstrated significant improvement from baseline to post-training assessment (*Z* = 21.0, p = 0.026) (Fig 3).

**Table 9:** Descriptive statistics of pre-training and post-training absolute self-estimation and cognitive performance measure for the experimental and control groups.

Measure	Experimental group		Control group		Quade nonparametric analysis of covariance (*p-value)*	Effect size (Cliffs Delta at 95% CI)
	Median (IQR) at pre-training assessment	Median (IQR) at post-training assessment	Median (IQR) at pre-training assessment	Median (IQR) at post-training assessment	
Absolute self-estimation (prediction discrepancy)	5 (2.25)	1.5 (2.0)	6.0 (4.0)	3.0 (5.0)	0.038	−0.52
Cognitive performance (CMT Recall scores)	14 (2.75)	18 (3.75)	14 (2.75)	12 (5.0)	0.02	0.62

## Discussion

Metacognitive abilities play a crucial role in effectively managing everyday tasks. Reduced metacognitive skills have been linked to an increased risk of cognitive impairments like Dementia. Additionally, the components of metacognition, specifically metacognitive monitoring and metacognitive control, have been reported to decline from young to older adulthood [27,28]. In particular, metacognitive biases, such as overestimation and underestimation of cognitive performance, along with implementation of inappropriate strategies during cognitively challenging tasks, have been reported to deteriorate as early as midlife [30,31]. In this context, the present study focused on developing and testing the feasibility of one such program, the MCST, which offered training for both monitoring and control related aspects for neurotypical MAAs. The following section discusses the salient elements of the developed training program along with key findings related to its feasibility.

The development of the MCST protocol followed a systematic process by employing mNGT approach [56]. Healthcare professionals in the field of cognitive sciences were involved in independent and separate rounds of mNGTs to select these strategy-related aspects and execution-related aspects. To identify strategy-related aspects, mNGT drew on insights from two different sources, incorporating ideas from NGT panelists and findings from the literature to enrich the data available for selection. This adaptability and ability to modify the steps of the traditional NGT method was particularly applicable for the present study and has been observed to be useful in other previous studies as well [75]. The structured consensus approach of the mNGT may have contributed to identifying key components of the MCST, which attempts to promote agreement among participants during idea generation by ensuring balanced participation from group members. This method has successfully informed key areas of intervention programs in previous research studies [76].

Regarding the strategy-related aspects of the MCST, the adapted metacognitive strategy ‘stop-think-plan-do-check’ received the highest ranking from the NGT panelists and, as a result, was taken into consideration. The STPDC developed during the mNGT through collaborative discussions among the panel members is inspired by metacognitive strategies reported in the literature, specifically the ‘goal-plan-do check’ [39,41] and ‘stop-think-plan-act’ [50] strategies. These two metacognitive strategies have been proven to effectively enhance cognitive performance among individuals with CCDs [39,41] and healthy older adults, respectively [50]. The overall framework of the STPDC strategy aligns with the recently identified IMPACT strategy [45]. In IMPACT, the initial steps of identifying goals and predicting performance parallel the ‘think’ step of STPDC. The subsequent stages of ‘plan’ and ‘do’ in STPDC align with IMPACT's ‘making plans’ and ‘acting’ phases. Finally, the processes of evaluating performance and experimentation correspond to the ‘check’ phase within STPDC. While both frameworks emphasize strategic thinking, planning, execution, and performance evaluation, the STPDC distinguishes itself from an additional ‘pause’ step inspired by the ‘stop-think-plan-act’ strategy. To successfully carry out daily activities, individuals must possess a clear understanding of their memory capabilities and predict whether they would be able to complete the desired tasks and if not, determine which strategies would enable successful task completion [73]. It is crucial to self-assess after completing a task in order to improve performance [64]. To gain such insights, one must take time-outs from automatic daily routines or cease automatic behaving [77]. The STPDC covers all these important elements and serves as a comprehensive guide to effectively carry out each of them, which would have resulted in the highest rating.

To increase the effectiveness of strategies, a platform that enables their application and practice must be established. The second round of the mNGT focused on identifying a suitable platform for participants to exercise the STPDC metacognitive strategy. The ‘client-centered personalized goal’ emerged as the most favored approach. This approach has also been utilized in studies focusing on metacognitive strategy training [44,46]. This method encourages participants, in collaboration with the specialist, to choose activities or tasks that they would like to use as a context for practicing the strategy. The emphasis is on identifying goals that hold personal meaning and relevance to participants daily life [46]. According to the goal-setting theory, when individuals set their own goals, they are more motivated and dedicated throughout the process. Moreover, this approach is linked to improved treatment outcomes [78]. The employment of such an approach was positively received by the participants in the present research, as they were able to readily recognize key areas where they sought greater awareness and improvement. They also reported that it was relevant to various aspects of their daily lives like successful completion of tasks at work, managing daily routines and appointments. The wide range of goals chosen by participants, in the present research, is the testimony of the success of this approach.

Progress monitoring is a crucial element of any intervention program, as it plays a vital role in assessing whether the participants derive benefits from the program. The second round of mNGT focused on identifying the methods to track the use of STPDC metacognitive strategy in daily life and the ‘daily-dairy’ method received the highest ranking from the panel members. The ‘daily diary’ approach has been previously recommended for assessing use of trained cognitive strategies in everyday life [50,77]. Using daily diary reports allows for short-term assessments within individuals to gauge everyday cognitive successes and failures. This approach is viewed to be more effective than assessments that replicate typical memory and cognitive functions when it comes to assessing individual behaviors, despite the potential for self-report distortions and biases in diary responses [77]. Therefore, the daily diary method was utilized to monitor both usage and effectiveness of STPDC across sessions in the MCST program. The daily diary reports of the participants in the present study reveal that all the participants found the STPDC to be beneficial in enhancing their understanding of their cognitive skills. Furthermore, six of the ten participants applied STPDC to additional goals not discussed during the sessions. These reports indicate the patient’s satisfaction with the strategy, their ability to generalize its usage, and motivation to incorporate it into various activities of life. The observed generalization effects suggest that the MCST program may have utility beyond the specific tasks chosen by the participants. It may have supported core metacognitive processes, such as error monitoring, self-regulation, and self-awareness, which participants were able to apply to various relevant everyday contexts. The generalization of strategy usage to broader contexts may indicate the potential transferability and applicability of the intervention. Moreover, participants reported that STPDC was associated with perceived improvements in everyday cognitive performance, self-efficacy, self-monitoring and self-awareness, while enabling them to successfully conduct their daily tasks and understand barriers and facilitators related to task performance. The majority also indicated proficiency in executing each step of the task across sessions, which may suggest that the intervention duration for the program was appropriate. The reflections received from participants through the daily diary reports exemplified below highlight the effectiveness of this approach in monitoring progress.

“It made me realize that certain tasks I am really good at. Certain tasks I struggle with. But I am able to reflect why is it I’m struggling. For instance, in learning this new language, I realized I am not spending enough time on rehearsal and practice that’s why I’m taking a long time to learn it. With remembering to-do list and face recognition of my clients, I realized I am able to remember things better than I had thought for myself” (P3)

The MCST protocol is comprehensive in all its attributes, as it borrows essential elements from existing protocols and offers a new, more relatable approach tailored to everyday scenarios and challenges faced during midlife. The adapted STPDC strategy encompasses the essential steps of a metacognitive strategy, ensuring thoroughness and efficacy. The client-centered approach enhances the protocol's relevance and meaning, making it more engaging for participants. Additionally, the use of a daily diary method as a reflective tool encourages consistent self-monitoring and personal growth. This combination of elements suggest that the MCST protocol may be practical and relevant for its users. This is supported by the responses from the feasibility questionnaire, which indicate that the design and implementation aspects of the protocol are both feasible and acceptable to the participants, requiring no further modifications. These findings preliminarily suggest the potential feasibility of scaling and applying the protocol in larger cohorts with minimal adjustments.

Apart from goal selection and daily diary reflection, the protocol was tested for its feasibility with respect to recruitment, retention, adherence, and acceptance, and to determine if it brought about changes in CMT immediate recall, absolute self-estimation, and self-perception of performance. The successful recruitment, retention and adherence rates indicate that the intervention protocol is feasible and well-received by the participants, providing preliminary evidence to support the implementation of larger scale randomized controlled trials to further evaluate its efficacy. The results also revealed significant post-training differences in all CMT measures between EG and CG. These findings suggest positive trends in metacognitive and recall performance among participants in the MCST group. These findings are congruent with other studies reporting improvements in awareness and cognitive performance following metacognitive strategy training [42]. The observed trends in the EG could be attributed to the monitoring-affects-control hypothesis [22], which proposes that improved metacognitive monitoring enhances metacognitive control, including the self-regulation of strategies. Moreover, the observed change in self-perception of performance measures suggests that participants are more adept at assessing both their correct and incorrect performance. Given that metacognitive deficits, specifically in memory domains, are known to precede cognitive deficits [52], these desirable changes in self-perception may empower individuals to more effectively recognize potential disorders. The findings not only demonstrated a positive trend in metacognitive skills of EG but also showed a positive change in the recall ability of the EG participants. The increased regulation of the learned strategies, self-assessment and awareness of task performance could have mediated cognitive performance, which is consistent with the strategy-deficit hypothesis and monitoring-affects-control hypothesis [23]. Another contributing factor may be the application of STPDC strategy in daily activities making it more relevant, functional, and sustainable. This approach may have helped individuals develop an awareness of their cognitive capabilities, leading to more accurate self-assessment, development of problem-solving skills for successful task performance, and fostering post-performance self-reflection. However, these findings should be interpreted as preliminary indications of the potential effects of MCST on cognitive and metacognitive skills. Further, given the non-randomized design and passive control condition, the observed improvements may also have been influenced by the non-specific factors such as participant expectations, cognitive engagement, therapist interaction, social participation, or Hawthorne effects. The observed positive trends warrant further investigation across a wider range of cognitive domains and highlight the need for large-scale randomized controlled trials with active control to rigorously evaluate efficacy and differentiate intervention specific effects from these non-specific influences.

The current research differed from other studies by not implementing the strategy during training sessions. Instead, the participants were simply introduced to the strategy and provided with detailed feedback on their experience in subsequent sessions. It is possible that neurotypical MAAs could benefit from this approach without requiring specific training within each session for using the strategy. Furthermore, the training protocol was found to be quick, easy, and not time-consuming for both, participants and SLPs, reaffirming its feasibility. Importantly, the present research extends the literature on metacognitive training by positioning it as a potential approach to support healthy cognitive aging. A recent scoping review highlighted that metacognitive strategy training protocols have majorly been implemented in the older adults or clinical populations with cognitive impairment [49]. In contrast, midlife represents a critical window of opportunity for preventative intervention, as subtle cognitive changes may begin during this stage despite absence of functional decline. Targeting metacognitive skills in midlife may aid strengthen self-monitoring, strategy regulation, and cognitive engagement before measurable deficits emerge. Thus, beyond feasibility outcomes, the current protocol contributes to the growing discourse on preventative approaches to cognitive health promotion.

### Clinical implications, limitations, and future directions

The developed protocol could guide healthcare professionals in cognitive rehabilitation as a primary preventive measure with healthy aging population. The current research used a methodical and scientific approach to develop the MCST protocol, identifying important areas for inclusion in metacognitive training programs. This could provide valuable guidance to clinicians and future researchers on the development and implementation of metacognitive training programs for various other demographics related to age, education, and socioeconomic status. Additionally, the promising results suggest that the MCST program has potential to improve cognitive and metacognitive abilities among MAAs and address age-related cognitive changes. Therefore, it could be a valuable addition in cognitive communicative interventions for addressing subjective memory complaints and functional cognitive disorders. However, the observed effects are preliminary and derived from a very small sample, reported only to highlight early trends. These findings now serve as a foundation for conducting well-controlled, large-scale trials. Furthermore, although CR was not directly measured, it has been conceptualized in the literature using static proxies (such as education, occupation) and dynamic indicators such as cognitive performance. Within this framework, changes in cognitive task performance may reflect dynamic processes that are theoretically linked to CR. While the observed change in cognitive performance may constitute one possible preliminary indicator of mechanisms associated with CR, such performance-based changes are indirect and should not be interpreted as definitive alterations to CR. Future studies are needed to examine the efficacy of MCST on cognitive reserve more broadly, beyond the preliminary indicators captured by the CMT.

According to the NIH Stage model, an intervention must demonstrate its highest level of effectiveness and be feasible for implementation with a large portion of the target population before it is considered developed. The current study concentrated on developing and testing the feasibility of MCST protocol in a small group of MAAs through a non-randomized design with passive control group that could limit causal inference and interpretability of the results. Although this limitation is acknowledged, it is important to note that internal validity concerns are generally less central to feasibility studies where practical insights into recruitment, intervention delivery and acceptability are prioritized. Furthermore, recent meta-analyses [79] in cognitive and behavioral intervention research have shown no meaningful performance differences between passive and active controls, particularly in feasibility and pilot studies, suggesting that non-specific influences such as attention, engagement, or expectancy may be less pronounced than traditionally assumed in this context. Nevertheless, the possibility of bias cannot be entirely excluded. Participants self-selected into the intervention group, and those choosing to participate may have differed systematically from controls in terms of motivation, engagement, or expectations regarding the intervention. Moreover, the passive control condition did not explicitly control for expectancy effects or other non-specific intervention-related influences. Therefore, the present findings should be interpreted as providing preliminary evidence of the feasibility, acceptability, and potential benefits of the MCST protocol, while its efficacy should ultimately be confirmed through adequately powered randomized controlled trials incorporating active control conditions. Furthermore, as the same version of the CMT was administered at baseline and post-intervention, the possibility of practice effects cannot be excluded. Although greater improvements were observed in the intervention group, future efficacy studies should consider alternate test forms or additional outcome measures to better distinguish intervention-related gains from those attributable to task familiarity. Further, the findings should be interpreted cautiously given the multiple comparisons conducted without adjustments, which may have increased the risk of Type I error. As the analysis were exploratory, future adequately powered studies should employ confirmatory approaches with appropriate adjustment for multiple comparisons. Further, the relatively homogenous sample recruited from a single setting may limit the generalizability of the findings to MAAs from diverse educational, cultural, and socioeconomic backgrounds. Future multicenter studies with larger and more diverse samples across different settings are warranted to establish the generalizability and broader applicability of the findings. Additionally, the program was developed primarily by SLPs, the absence of broader interdisciplinary input such as neurologists, neuropsychologist, clinical psychologists, may have limited the scope of perspectives incorporated into the protocol. The inclusion of both subjective and objective measures is a notable strength of the study. While this study only assessed effects on short-term memory related outcomes as the aim was to mainly assess the feasibility, future research in this area can explore its impact on other cognitive domains such as working memory, prospective memory, and executive functions. Although promising trends have been observed, this study did not investigate the long-term maintenance effects of these positive changes. Studies examining the long-term benefits of MCST protocol are crucial. Future studies could also investigate whether the MCST protocol is feasible and effective across various demographics. The present study assessed intervention acceptability using a questionnaire-based method, which may have been susceptible to ceiling effects. In future iterations, we intend to incorporate a qualitative approach, such as semi-structured interviews, to capture more nuanced participant perceptions. Further, the intervention acceptability in the present study may have been influenced by the perceived relevance of daily diary goals to participants everyday lives. Future studies could examine how varying levels of goal relevance impact perceptions of intervention acceptability.

## Conclusion

The present study aimed to develop a metacognitive strategy training protocol for MAAs aligning with Stage I of the NIH Stage model of behavioral intervention development. Stage IA, which involved the development of the MCST protocol using mNGTs, resulted in identifying STPDC metacognitive strategy. The execution-related aspects identified for the protocol included client-centred personalized goals for goal-selection, daily diary reports for tracking progress, and seven-session protocol (1 pre-, 5 training, and 1- post-assessment session). Following this, the MCST protocol tested on MAAs (Stage IB) revealed a significant improvement in absolute self-estimation and cognitive performance score of participants in the EG compared to CG. The self-perception of performance rating also significantly enhanced following training. Furthermore, all participants were able to identify a specific goal to focus on and found the STPDC beneficial in aiding their comprehension of their cognitive skills. Their proficiency in utilizing metacognitive strategies improved over the course of the sessions. Six out of 10 participants applied the metacognitive strategy beyond the initial goal discussed in training. The participants expressed that STPDC strategy was advantageous for improving cognitive performance, self-confidence, self-assessment, and self-awareness, enabling them to effectively carry out tasks while recognizing obstacles and factors that assist task completion. Participants found the design and implementation aspects of the training program to be acceptable. The objective findings on the CMT and participants’ subjective reports suggest that the MCST protocol has the potential to improve certain aspects of cognitive performance and related metacognitive functioning in aging adults. These findings also provide a promising foundation for future investigations examining whether metacognitive strategy training contributes to broader theoretical constructs, including cognitive reserve and healthy cognitive aging. These findings lay the groundwork for further evaluations aimed at assessing the efficacy of the MCST protocol in larger-scale controlled clinical trials.

## Supporting information

S1 Appendix Interview probe questions and response ratings. (DOCX)

S2 Data Raw data of absolute self-estimation measure and cognitive recall scores of each participant. (XLSX)
