# Supplementary material for: Developing Midlife Metacognitive Strategy Training (M-MCST) program: A feasibility study with middle-aged adults to evaluate the design, implementation, and impact of a novel program in fostering healthy cognitive aging
Source: PLoS One. 2026 Sep 25;21(9):e0358223. doi: 10.1371/journal.pone.0358223 (PMC13614589; doi:10.1371/journal.pone.0358223)
Supplement: Supplementary file 1 [file pone.0358223.s001.docx]

**S1 APPENDIX**

***Interview probe questions***

1. General awareness question: Did you make any errors in the task? What were the errors?
2. Symptom-specific awareness question: Did you experience any difficulty in recalling the pictures? (Question is specific to the cognitive symptom observed)
3. Task-specific awareness questions: Did you experience difficulty in recalling “*state the example*”

***Response rating***

| **Rating** | **Self-perception of performance** |
| --- | --- |
| 1(good awareness) | Client is able to accurately reflect if or not errors are made. When errors are made, client acknowledges cognitive difficulties with general questions and can provide specific examples of all errors made |
| 2 | Client acknowledges cognitive difficulties with general questions and can provide specific examples of almost 50% of errors made  OR  Client suspects (or doubts) of errors in performance when there were no errors observed (Ex: I may have made an error, I don’t know) |
| 3 | Client acknowledges vague difficulties with general questions but is unable to provide specific examples  OR  Client identifies errors in performance when there were no errors observed |
| 4 | Client acknowledges difficulties only with questions directed at specific cognitive problems or symptoms  OR  Client acknowledges successful performance only when provided with feedback |
| 5 | Client acknowledges difficulties only with task specific-questions |
| 6 | Client blames errors on external sources, identifies problems with a task or physical deficits |
| 7 (unaware) | Client doesn’t acknowledge any errors, challenges, or difficulties within the task |
